# Stone age diseases and modern AIDS

**DOI:** 10.1186/1743-422X-5-93

**Published:** 2008-08-07

**Authors:** Arthur L Koch

**Affiliations:** 1 Biology Department, Indiana University, Bloomington, IN, 47405-6801, USA

## Abstract

The great advantage of being a sexually transmitted disease is the ability to survive and specialize solely on a host species that is present in low numbers and widely distributed so that contact between infected and uninfected organisms by chance is rare.

Pathogens of a sparse, but widely distributed host species, must either: i) have an alternative host; ii) be able to survive in a dormant state; or iii) be non-destructive to their host. For the pathogens of a diploid there is a particularly effective strategy, that of being sexually transmitted. Then the hosts' themselves transfer the pathogen.

## Sexually Transmitted Diseases

The advantages of this mode of pathogenic transmission are clear, but the great disadvantage is that evolution has now produced many very sophisticated mechanisms in the host to block pathogens of various origins and specialties from entering and growing effectively in the sexual apparatus of the female mammal.

Thus, the biological problems that STD mechanisms face are because of the countermeasures against the pathogens that are due to evolution of the host. However, the mechanisms that have developed that protect the fetus from the host's immune system do help the STP.

### Problems with the STD approach from its point of view

There are two major problems with the STD strategy: First, a sexually transmitted disease must be (at least relatively), a 'gentle pathogen' or a 'prudent predator' and remain as such (i.e., it may only evolve towards greater virulence slowly, or not at all). Secondly, such a pathogen must, to a large extent, be able to avoid destroying its mammalian host's offspring before the child grows to a sexual adult stage. Both factors would seemingly be countermanded by the immediate selective advantage of being more virulent and producing more propagules.

### The Vertebrate's Female Reproductive Tract (FRT)

The immune system of the mammalian sexual system has many wonderful properties because of the many problems that the Female Reproductive Tract (FRT) faces. The immune system of the FRT is nonpareil for its diverse abilities to solve these problems. Possible pathogens include a large range of viruses, bacteria, and protozoa. Mostly these are destroyed or eliminated by the FRT. Additionally, when there is an embryo: this propagule is foreign because half its genes are not derived from the mother. The mechanisms of the FRT must not operate against it. Although, it is a foreign object like all the pathogens of the host, this particular entity is to be protected.

### The Fetus is Special

Consider a Sexually Transmitted Disease (STD) from the pathogen's point of view: it must successfully pass into the female sexual tract, possibly into her body, and into the next adult generation of hosts. So the STD faces a quite different fate than other kinds of pathogens.

### Advantages of Vertical Transmission

To reiterate the virtue from the pathogens' point of view, the STD strategy is favorable because the pathogen is propagated to new hosts without entering the environment. Thus, the infection of a new host is expedited by the old host's behavior in searching for mates, and thus many mechanisms used by other pathogens for transmission are not needed.

### Complications of the Many Roles in the Female Sexual Tract

The FTR is a complex of organs: ovary, fallopian tubes, uterus, cervix, and vagina. They have many physiological aspects that are quite different from each other and each is essential for the propagation of the host species. They, of course, all do act in a way favorable for reproduction, but unfavorable, at various degrees, to pathogens. But the STD's are treated quite differently.

### Consequence of the Absence of an Immune System in the Fetus and Newborn

The lack of an effective immune system in the fetus (even in the presence of maternal passive immunity) and in the newborn (even given mother's milk with colostrum) implies that the STD pathogens might propagate *in utero *even though the mucosal immune system of the female sexual tract may be able to reduce the danger of infection and disease to many disease agents to the offspring.

### Importance of Protecting the Fetus, the Neonate, and the Pre-pubescence Child

In the long run, with many ins-and-outs, the current situation is favorable for the STD pathogens because the long term interest of the host species is that the young members of the host population grow to become sexually mature and this provides hosts to become infected and propagate the pathogen.

## Stone age life

When populations of humans arose and expanded and emigrated in the early Stone Age, the problem that pathogens would have of how to propagate and surviving in this sparse host population became critical. Cave-dwelling primitive humans, 30 thousand years ago that lived in small well-separated tribal populations would have had quite different diseases than humans have today. Workable disease possibilities are few but would include, sexual transmitted diseases. However, this required that the pathogens were 'gentle' to their host. Other possibilities for these pathogens are either: acquiring and using an ability to remain dormant for long periods of time or of being able to infect a ubiquitously occurring alternative species. These latter two possibilities will not be examined further here, but they do occur in nature. The option of being 'gentle' to the fetus is the main topic here with respect to STDs, but a further aspect of this will also be presented; this is that an STD of a mammal will face the special problems of preventing the destruction of the fetuses and neonates of its host because these are needed for its own propagation.

### The Advantage of being an STD in a Sparsely Populated World

There is an essential feature needed for a successful infectious disease of social animals that are distributed in dispersed state in individual groups with small numbers. This is to have a way to spread from social group to social group. In sundry diseases [1-4], this is done by forming long-lived spores, or passing through intermediate hosts or vectors, or being carried by various animal and insect vectors over significant distances. Another very effective and quite successful way, however, is to use a sexual mode of transmission, which depends on the host actively interacting with spatially remote populations of hosts. In general, many STD pathogens are not long lived within the environment outside the host's body. In addition, they are usually highly specific so that they do not have an alternate host (except only very rarely); consequently before the invention of the hypodermic needle, these STD diseases usually had only a sexual mode of transmission within the individuals of a species [5-9]. STDs must be extremely common since there are many viruses that are quite mild (Hoeprich *et al*. [1]) and I assume that there are many more such agents that are so mild that they have not been detected.

Depending on the degree of interaction between social groups, this strategy would be unsuccessful if the pathogen was highly lethal and the host populations widely distributed, since they would be eliminated from a local population by destruction of subpopulations and usually not be successful in reaching neighboring communities. This is the way, I presume, that ebola was in rural Africa in the past: it arose from some animal into a human social group, destroying that group, then the viruses were eliminated, ending that particular episode. Consequently, any successful STD disease would need to be as 'gentle' as possible while still retaining its infectiousness, if it is to be effectively transmitted to other hosts during rare encounters between groups when population levels are low and well separated.

A distinction needs to be made from a mechanism that differentiates survival of a 'gentle pathogen' from the evolution of the pathogen as the results of group selection. Group selection occurs, for instance, if there is the possibility that some pathogen may contain or acquire a gene that confers some advantage or level of resistance to the pathogen. This gene and its population would then be selected because pathogens with that gene would prosper and become dominant.

The long-term persistence of a disease among sparsely distributed social animals would depend on the particulars of the social interaction between groups. Passage of a STD from group to group is aided by the social behavior of the hosts, which may institutionalize transfer. For example, the social groups of many kinds of mammals' centers around a single dominate male or a female of a bonded social group [9-13]. Consequently, other (usually younger) males or females of a variety of species are ejected from their natal small groups. Sometimes these may become the dominant in other groups. However, they may incidentally carry STDs with them.

In chimpanzee troops and other primates living in groups there is often the exchange of young females between groups as the females reach sexual maturity. In many cases, these females are forced to leave their original group. Possibly this same strategy was practiced by early human groups. After the female chimpanzees emigrate, they are taken into neighboring colonies, spreading STDs. Not only is this mixing of populations known from direct observation of various non-human primate species, but it also can be deduced by the smaller degree of polymorphism for genetic markers exhibited in the male population of a clan of chimpanzees relative to the females in the same group.

This process of exchange may serve the primate species very well by limiting the effects of inbreeding. The explanation of these behaviors by geneticists and sociobiologists is that consanguinity is bad for any species (except obligatorily selfing-organisms, like Mendel's garden peas that do practice incest habitually). Whatever the validity of this explanation and how the custom arose, sexual mixing occurs between social groups as an institutionalized process even in the absence of prostitution, rape, and war. Even if these violent transmission events between host groups may be fairly infrequent they, and the less violent, custom-justified, mixing between social groups are essential to the STD's way of life.

### Diseases in primitive humans

A sexually transmitted disease gains most of the advantages of vertical transmission in not needing to be transmitted through the environment. Moreover and very importantly, it can survive in sparse populations because of its host's sexual proclivities. In these two sentences the essential elements for a parasite (especially a virus) to survive by infecting humans as its only host in the Stone Age have been spelled out. In such circumstances it was necessary for a pathogen to be able to compensate for the low population density and sporadic distribution of its host. During the tail end of the most recent Ice Age, the human hosts survived in small, mostly isolated, groups that figuratively chased mastodons and other big game as a group effort.

Those pathogens that depend on humans as a resource faced a much different problem after domestication of plants and animals than during the hunter/gatherer Stone Age culture. Tuberculosis did occur in people in the ancient Egyptian empire, but by then the population was locally quite dense and transmission through the air from person to person in large populations became efficient. The problem of pathogens became still quite different during the Industrial Revolution and in the medically sophisticated world of today. Possibly, at the time of Christ the world population was a thousand-fold larger than during the Ice Age, and probably, the world population of humans has increased more than a thousand-fold since. Today, with the world human population increased, with transportation easier, with higher local population densities, with more rapid migration, and with mixing of humanity taking place at an unprecedented rate the situation is entirely altered. These changes must result in a great increase and alteration of the spectrum of diseases, and particularly of communicable diseases that are transmitted effectively between people that are crowded closely together. For this reason, it can be argued that the major diseases of Stone Age humans were largely STDs (or with those pathogens capable of remaining dormant or propagating in other hosts) and the typical major infectious epidemic diseases experienced in the early Christian era and afterwards were not. The aftermath of hypodermic needles, jet planes, and other modern inventions are that the spectrum of diseases has become much different still.

The two keys of the matter are: first, that many of today's STDs have had a long association with primates, including humans, and have had an opportunity to modify their hosts; and in return, the pathogens have been modified by their host's biology. Second, the same group-group interactions, as in the hunter/gatherer cultures described above, apply to other primate populations living in the wild today. So we can assume that STDs specializing in particular species have been around for a very long time in human and other primates populations, and that they should generally be 'well-tuned' to their host species' particular sociality. Furthermore it can be assumed, that only occasionally will such adapted STDs be dangerous or lethal to their specific host species, or at least to a fraction of the individuals of that population. To the degree that HIV has recently entered the human population or more specifically into the population of a some times medically treated, modern, industrialized, jet age men and women, it is now in an unfamiliar milieu and is especially dangerous (Gilbert [2]).

## The strategy of cave man's diseases and those of modern primates

All STD's of cave men (I presume) and today's wild primates face the same general problems. But here we will be more specific and consider the problems of the retroviral STDs of monkeys and man [10-14]. Now let us match the retroviruses of primates to the design criteria for a STD pathogen of sparse populations. Point by point these criteria are met by viruses abundant in non-human primate populations in Africa today, such as SIV (Simian Immunodeficiency Virus) that is resident in the African Green Monkey. Of course, SIV may be more virulent when transferred to different monkeys; e.g., to the geographically distant Asian ones, than the species from which it was initially isolated. Of course, SIV would be virulent in animals that happen to have a defective immune system. The general point is that the small deviation from its behavior that has been optimized for a retroviruses' life in its native host now may lead to catastrophic problems in the variant host, whether it is the human, the Asian monkey, or the compromised host.

It is assumed that HIV emigrated to humans relatively recently; see [15-21] (and it is thought that 1930 was in time of transmission to man from primate in the Belgian Congo [20]. This virus and its new human hosts have not had a chance to adapt genetically to each other though there are some indications that there have been developments in this direction (Ewald [21]). One of the mismatches between the disease and its human host that most affects disease virulence is that the immune system after HIV infection of the human being deteriorates in 5 to 15 years; this same period would be unimportant to non-human primate populations in the wild because they have shorter mean life-spans and maturation periods and may generally die of other causes before they lose their effective immune system. On this basis, it may be that the changes needed to re-establish the 'gentle' parasite mode in the new human hosts of SIV are minimal: For example, just a shortening of the human life span to that of the turn of the nineteenth century level would do. A disturbing, but realistic, suggestion of a change that would certainly make HIV infection relatively more 'gentle' is a general re-emergence of life-shortening infectious diseases, such as tuberculosis. There are many other diseases that may erupt as the antibiotic era closes, and with the loss of efficacy of antibiotics, the human life span may decrease dramatically and the immune system may survive to the end of the life-span and outlasts the shortened length of life-span of its host as the result of deaths from other causes than just the effects of the AIDS. Then there would be a lower proportion of individuals with signs of ARC or AIDS. Changes of the other kinds will be suggested below that might increase the longevity of the immune system in an AIDS-infected individual in a long-lived population, but other possible changes might decrease it.

## The mucosal immune system

Of the many kinds of immunological responses, the ones that function at mucosal surfaces are most relevant to STDs during their transmission from individual to individual [22-40] The feature relevant to STDs epidemiology is that the female sexual tract is the right place for ways to prevent new infections.

## Immunology of the female reproductive tract

In order to be propagated, a sexually transmitted disease needs to grow and be transmitted through both the female and male reproductive tracts to new hosts. On the other hand, particularly the female productive tract has evolved mechanisms to overcome pathogens that enter it. However, an important additional component for the disease is that the STD's somehow needs to ensure that the embryo, fetus, and child of its host do not die, but grow to sexual adults so that they too can serve as habitats for STD to grow. "Ensure" is too strong a word as long as the pathogen is only successful in attacking a small portion of the host population.

Evidently, there are mechanisms contributed from both the human and the virus to protect the juvenile against destruction by the HIV, beginning at conception and continuing on to near adulthood. This is because otherwise the fetus and neonate would be destroyed before leading to an adult that can receive and propagate the virus.

The complex immunological processes within the pregnant female are indeed very sophisticated. This has been well studied because the interest in this question is high since this may be a possible basis for the prevention of the spread of AIDS. It was, and is hoped, that immunological ways could be developed. However, here the interest is to try to understand the biology to the host-parasite interaction from the point of view of the disease process. There must be some blockade to transmission extent now because even though HIV infection through sexual contact of adult-to-adult is efficient, there are some fetuses and children that are not infected or killed by the infection, but grow up to become adults to be reservoirs for growth and propagation of the virus.

A strong indicator that HIV is usually prevented from infecting children perinatally is that while the majority of children born to HIV-positive mothers are not infected, they almost all carry maternal antibodies against HIV 41 (McWhinney, Pagano, and Thomas. [40]). Presumably they had them when *in utero*, but either had not become infected or had overcome it.

Going even farther out on a limb, one can imagine that the mucosal immune response could protect the baby during the birthing process [41]. This might be accomplished by a strong IgA or T-cell response, or by an IgG response that is delivered through the placenta [42]. If this were possible in terms of the host's immunological repertoire modulated by some stimulatory action of the pathogen, this would increase the long-term fitness of the STD. Such a situation would lead to continued selection for such genetic variants of the host and/or in the STD. Then of course, through successive generations of virus and host in an environment with high levels of other pathogens, these developments would 'tune' the growth of the particular retrovirus to a particular primate species. These changes may lead to effective prevention of infection due to secondary pathogens or due to superinfection of the resident sexual disease by other copies of itself, and would have a long term (and incidental effect of maintaining the host population). Such selection would lead to corresponding changes so that the pathogen becomes milder to the host as a consequence of induction of greater immunogenicity against the resident pathogen and would be beneficial to the host because the first pathogen would prevent other, possibly more dangerous pathogens, from entering and destroying it.

The converse possibility is that the pathogen causes deleterious effects due to stimulation of the immune system. In many cases these effects are significant, but generally not lethal.

## Human antimicrobial factors

### Defensins

There are a number antimicrobial factors made by the mammal that protect it against pathogens. These include many defensins. Of these, human β-definsin-1 is especially to be considered because of its location in urogenital tissues (Valora *et al*., [43]).

Retrocyclins that are θ-defensins and their variations have important effect against anthrax [44]. Also, some forms of θ-defensin and α-defensin have protective effect against HIV-1 [45,46]. But the latter paper did not find an effect on the mother-to-child transmission. Quinones-Mateu *et al*. [47] show that human epithelial β-defensins 2 and 3 inhibit HIV-1 replication. Aono *et al*. [41](2006) find that in bovines the bovine β-defensin-1 acting against *E. coli *and is present in teat mucosa, vagina, ovary, and oviduct. These references, and also MasCasullo *et al*. [48], allows the possibility that I have not seen explicitly suggested that these substances in the (female reproductive tract) FRT act to prevent the embryo and fetus from infection by a virus that has infected the mother. Furthermore, that they, except for the α-defensins, have a role in the partial protection of the newborn from infection by way of the mother's milk.

### Secretory leukocyte protease inhibitor

The secretory leukocyte protease inhibitor (SLPI) may also be a factor that acts in prevention of HIV infection. It is known that salivary gland tissue may have a role in suppressing transmission by the oral route. Wahl *et al*. [49] were able to account for the rarity of oral transmission even though there is HIV in oral secretions. Once inside the oral cavity, HIV is exposed to antiviral levels of SLPI, and their data suggest that this may greatly impede infection. An extrapolation, for which there is no data, could be that HIV might stimulate the synthesis of SLPI. A further extension from known facts is that it may also takes place in the genital tract and prevents secondary infection of HIV viruses, or variants, or a broader class of STDs [50]. These suggestions could be tested.

## Variable stimulation of the immune system of primates under different conditions

The long-term survival of the retrovirus pathogen in vertebrate hosts depends on proper balance of the pathogen's activity versus the hosts' immune systems. A possibly critical factor of difference between modern humans, the Stone Age man, and the modern non-human primate needs to be recognized here. Some of the possible differences would make the response greater or weaker, and consequently could support or conflict with the hypothesis proposed here. This balance could depend on the variety and variable extent of immune stimulation in general and is, in addition, to the responses of these host species to their particular varieties of retroviruses. The stimulation of the immune system, variety of stimulations, and the age dependency of individuals exposed to other antigens before they are challenged by viral antigen do vary (see for example, Esquerré M *et al*. [50]; Walker *et al*., [51]; Chirmule *et al*., [52]; Rabin *et al*., [53]; Miedema and Klein, [54]; Wolensky *et al*., [55]). There must be marked differences in the sensitivity to antigenic stimulation of the members of primate societies in the wild, of the humans in developing countries, and of the humans in developed countries. Either too much, too little, or the wrong kind of stimulation may mean that the immune system will be either over or under active, because of the effect on the mucosal part of the system and, this is significant for the theme of this paper. A slight variation may eliminate the pathogen or fail to prevent new pathogens from entering and supplanting the original one. The immune system may be secondarily modified by destruction of certain specialized T-cells in which HIV or SIV pathogen are present. As a speculative example, young female chimpanzees engage in some sexual activity for some time before they can become pregnant. If the female has been infected with SIV for several years before she becomes fecund it would not be surprising if she had slowly developed a range of mucosal immune responses that would have a protective effect on her and/or the developing fetus. This effect might not occur if she had been infected more immediately before pregnancy. Some aspects of the AIDS disease process in humans are certainly similar to autoimmune diseases and the destruction of cells of the immune system bearing viral or similar receptors is well known. All that is being suggested here is that such phenomena need be only slightly altered to give virulent disease instead of an innocuous one in the not quite well adapted AIDS-human pair from those of well-adapted long-term retrovirus-primate pairs. In the current worldwide AIDS epidemic these could affect the timing of symptom onset and the severity of debilitating disease.

At an earlier time when our blood supplies were contaminated, hemophiliacs were very likely to receive the AIDS virus and become HIV seropositive. A significant point is that their time to conversion to ARC and to fulminating AIDS was 90% longer than other non-hemophiliac seropositive persons at that time Darby *et al*. [56] in 1995. One possible reason is that the medically treated hemophiliacs were being continuously challenged and immunized against a large variety of other substances that were present in the various blood transfusions that they regularly received. An additional factor concerning the AIDS disease at the beginning of the world epidemic is that when hemophilia patients were initially infected by transfusion, the disease would have been started with a much larger number of viruses than that transmitted either by usual or unusual sexual practices or by a drug addict's re-used needle. Consequently, their immune system was stimulated in a way that the immune systems of normal people infected by sexual contact are not, simply because the intensity of the immune challenge was greater. These facts raise the possibilities that African green monkeys are immunologically equivalent to human hemophiliacs receiving blood transfusions. As a result, an African green monkey from which SIV can be isolated might naturally have an especially effective immune system and be able to continuously destroy a much larger proportion of retroviruses and thus limit the viremia. This could mean that the animal would live for a long time before it might become immunologically deficient. In any case, it will be interesting to see the disease progression in fresh hemophiliac patients that were infected by sexual transmission and not by transfusion either while they are receiving recombinant clotting factor without these multiple sources of diverse immunogenic stimulation or with them.

Immune reactivity can depend on the presence of other pathogens: Infection with mycoplasma, Herpes, Epstein-Barr, and several other viruses may affect how HIV infection leads to an HIV-immune deficiency state. With co-infection of such viruses, it is likely that the time at which AIDS erupts may be sped or slowed in either the human or monkey. This raises the possibility that a different spectrum of viruses and other possible diseases might influence the course of a retroviral disease. A sometimes-symptomless retrovirus that suppresses the development of debilitating immune deficiency might do so differently when infecting a different host species. On the contrary, other pathogens may trigger the emergence of the AIDS virus from the host's chromosomes. These assorted immunological events could greatly affect the life history of a retrovirus in any new primate host. Consequently, it can be argued that the AIDS virus may well have been adapted to be 'gentle' and un-obstructive in its old host, however, at present, those strategies do not work as well in humans because the human pathogens are different than the non-human primate. Because of our social organization, our life expectancy, and our antigenic environment, the outcome of this parasitism might well be quite different.

A very striking observation was made fourteen years ago [57,58](Ho *et al*., 1995; Wei *et al*., 1995; but see Pang S, [59] and see Wain-Hobson, [60] and Miller, Antia and Levin, [61]. The major conclusion is that the HIV viruses grow very rapidly and are very rapidly destroyed by the apparently healthy, but HIV positive individuals. It leaves unanswered how much of this destruction is due directly to the action of the host immune system versus the action of the host when modified by the virus or responding in other ways.

The possibility is that there are protective mechanisms implemented by the resident HIV. A finding that is relevant concerns an individual who became infected with only one strain even though two different varieties of HIV were transfused simultaneously into him [62].

## Epidemiology of AIDS and the 'rapid' change of HIV

By comparing the epidemiology of HIV-I and HIV-II in their respective parts of Africa, Ewald [21] has made the case that the diseases vary in the virulence/gentleness scale in a way that is correlated with the number of sexual partners and the group's societal norms. This would be predicted by a game theory type calculation on the bald assumption that the virus was omniscient without providing any suggestion of how it got to be so. Accepting his ideas and facts as true, we can only be amazed by the speed with which the virus can apparently change its strategy and optimize the course of infection to the new conditions present in a new host. I think that this means something more significant. As an alternative to *de novo *evolution, I suggest that this apparent rapid adaptation is consistent with the hypothesis that STD retroviruses have been exposed over long evolutionary times (in terms of millions of years) to fluctuations in the behaviors of a series of hosts (or a host under a series of very different conditions) and have developed and retain a genetic repertoire to be able to achieve response to variations in the host. From the molecular point of view this could allow them to track and respond to the behavior of their current host and conditions. Although the genetic sequences are now known, the roles of the gene products are not fully evident and some of the regulatory genes may have functions over a long time period of years and centuries.

## Why mammalian STD pathogens must protect their new generation of pathogens from their own lethal action

A careful strategy must be maintained for long term persistence between the STD virus spreading from adult-to-adult and in injuring or killing the children of an infected host. The biology of pediatric AIDS is reviewed in Pizzo and Wilfert [63], Remington [64], Roizman [3], Sweet and Gibbs [65], and Kaschula [66]. Teleologically, the virus must spare the host's young in order that they can be used as a resource in the future, but that necessary-truth provides no mechanistic way to implement such a situation. HTLV-I (see below) seems to have achieved this balance by infecting most offspring of infected mothers but having the virus grows so slowly that a child's life before puberty is virtually unaffected. Such slow viral growth permits propagation of the virus to virus-free individuals within a society in which sexual contact is frequent and avoids the usual destructive effects of vertical transmission from mother to child. The AIDS viruses, HIV-I and HIV-II, do not seem able to avoid injuring the children after infection occurs. But still a large proportion of the young of infected mothers do not become infected – more than 60% and in some estimates as much as 85% for HIV-1. Some infants, born infected, clear the HIV virus (Clerici *et al*. [67]; Roques *et al*. [68]).

An infectious disease spreading to the next generation by vertical transmission has advantages over pathogens disseminated in other ways. The main advantage is that the disease organism never has to survive in the environment outside of the host. Survival of a pathogen outside of its host in air or water takes special mechanisms. Although the fetus is a convenient and a built-in susceptible host, the vertical process in vertebrates has a special complexity beyond that present in the cases when the host grows by binary fission as bacteria do. This is because the animal host gives birth to an immunologically ill-equipped neonate that may not be able to survive due to damage caused by the pathogen. This is a critical problem for an STD's survival strategy because they generally have no alternative propagation strategy such as survival in alternative hosts or persisting in the environment.

Because the newborn offspring do not have a full immunity system the result is often catastrophic. This is what happens when some pathogens, such as Herpes Type II, Rubella, or Cytomegalovirus infect an embryo, a fetus, or a newborn child. In these cases, without a fully protective immune mechanism, the infection may be destructive or lethal to the offspring and is much more severe than if the pathogen infects an adult that has a responsive immune system with an immune repertoire already in place.

Therefore, in many cases a would-be pathogen cannot effectively persist via pure vertical transmission simply because the pathogen cannot depends on its host immune response system to bridle its growth. Viruses like Herpes, Rubella, and Cytomegalovirus that go across uterine, vaginal, and placental tissue are frequently dangerous to the fetus, but of course these viruses survive because their primary means of spreading through the population is from an adult individual to an adult individual and are not dependent on vertical transmission. Destroying a few children apparently does not upset the growth success of these viruses. However, from the viewpoint of a disease that has elected to only use a non-virulent STD strategy, going directly from mother to baby could be expedient, although destructive. This must somehow be avoided, and it apparently is often curbed by successful pathogens.

There may be a number of factors involved in the AIDS case. One is that the AIDS virus is just not very infectious or long-lived. Some suggestion of this comes from the fact that AIDS is not transmitted by way of bloodsucking mosquitoes or efficiently through punctures of health care workers with contaminated needles. Presumably, in the mosquito example, this is because the virus does not last long enough between successive blood meals of the female mosquito. Additionally, it may not be transmitted because infection requires large inoculums. Similarly for the second example, the fact that HIV is quite seldom transmitted through needle sticks to health care workers, is possibly because most particles are inactive and many viable particles are needed to cause infection of a healthy health care provider.

While these could be trivial or based on molecular biological necessity, I suggest that the virus has been selected by evolution to extend these characters. Evidently, this is the characteristic of particular viruses, but it also may be due a human or primate characteristic. This can be if mankind has habitually, over the eons, been exposed to retrovirus type pathogens that are not AIDS. For both virus and host, their fitness is increased in not permitting infection of the still immunological-incompetent neonates. It may be that the low infectivity of neonates is because of some molecular biological or biochemical limitation or necessity, but in the retrovirus case it has been especially extended. This can be argued since there are diseases that are transmitted very efficiently by mosquito bites and by limited blood-to-blood contact (such as Hepatitis B). So I suggest that the HIV retrovirus is poorly infective due to innate biological mechanisms evolved to favor the long-term goals of its survival as an STD. This is a prediction of the model proposed here that would have aspects that could be experimentally pursued.

Although I will discuss below how the retrovirus human T-cell lymphotrophic virus I (HTLV-1) avoids this difficulty by known mechanisms, just how HIV perinatally infects only a portion (15 – 30%) of children (Scott [69]; Cotton [70]) and not more is not clear. Women that acquire HIV after delivery have a higher transmission rate to their child by breast-feeding than do women previously infected. We have also a hint because reducing the viremia by zidovudine (AZT) treatment before and during the delivery process has been shown to reduce the transmission to the child (Connor *et al*., [71]; Spector *et al*. [72]: Rouse *et al*., [73]; Brossard *et al*., [74]) and now is routine. However, there are reports of several children that have been initially infected and apparently cleared themselves of the infection (Bryson [75]); this makes it difficult to compare different studies.

## The coping strategy of HIV, HTLV-I, and HTLV-II with their hosts

HTLV-I and HTLV-II are both retroviruses of the subclass oncornaviruses that propagate primarily in human T lymphocytes. The former parasitizes the CD4-bearing helper T cells and the latter the CD8-bearing cytotoxic T cells (See Anderson [76]; Höllsberg and Hafler [77]; Wiktor and Blattner [78]; Blattner [79]; White and Fenner 1994 [5]). HIV is a retrovirus of the subclass, lentivirus, and both it and the oncornaviruses incorporate their reversely transcribed double-stranded DNA into a chromosome of a human cell as the heart of their survival strategy. Both classes of virus infect only a particular human cell-type and only a cell type that continues to divide. Nerve cells and kidney cells are not appropriate host cells because in the adult they divide only very occasionally and therefore the equivalent of the prophage state would never be adequately created or propagated. Also an inappropriate cell type would be a relatively rapidly growing epithelial cell because while the stem cell remains, the sister cell is sloughed from the skin or into the intestine and does not remain and be propagated within the body.

The oncornaviruses, but not the lentiviruses, have a growth pattern that allows both effective vertical transmission and horizontal transmission to new hosts. The viruses are passed vertically to the neonate, although not with high efficiency and not in a way that causes childhood death. They are also passed as a STD between sexually active individuals. In addition, in the modern world these viruses can also be passed via needles. Before extensive movements of peoples of the world population, both HTLV viruses were geographically restricted in distribution. Although HTLV-I is now found world wide, it was and is highly abundant in southern Japan and the Caribbean. It is also present in South America, west and central Africa, India, Melanesia, and Iran. There is evidence that similar viruses inhabit non-human primates. HTLV-II is now highly prevalent in intravenous drug users, but is (and presumably was) abundant in the Guaymi tribe in Panama and, more generally, in Amer-Indians.

The strategy of both HTLV viruses in the absence of IV drug usage and rapid movements of peoples was very close to the 'gentle' pathogen persuasion. Both viruses persist with little damage to their host by replicating very slowly while being passed vertically or between adults as an STD. Being poorly transmitted from cell-to-cell and individual-to-individual favors the 'gentle' character, but another feature is that they have mechanisms to cause the hosts T-cells to replicate. Thus a syndrome, similar to that of AIDS does not occur, resulting from depletion of T-cells. This allows more opportunities for the virus to be transmitted between humans. They are poorly transmissible and transmission from male to female is rare and the transmission from female to male is very slow (at least for HTLV-I). Though it is passed presumably in the same three ways that HIV is passed to the neonate (i.e., in utero, perinatally by blood-to-blood transfer, and (predominately) postnatally via mother's milk). There is little damage to the child because the virus grows so slowly. Thus the immune system has time to develop and respond to these viruses and is even aided by the increased growth of T-cells. Both viruses are almost perfect 'gentle' pathogens. However, the current longevity of the human host is sufficient to cause problems (see White and Fenner, [5]). For example, there is the occasional generation by HTLV-I of an adult T-cell leukemia (ATL). Less frequently T-cell chronic lymphocytic leukemia, non-Hodgkin's lymphoma, mycosis fungoides, and Sezary syndrome arise. HTLV-II has not been adequately studied, but appears to be even more 'gentle', and only very rarely causes certain diseases: i.e., glomerular nephritis and HTLV-associated myelopathy.

Molecular biological information is mainly available for HTLV-I, but the studies of the *rex *and *tax *genes are relevant to the trick that this virus has of alternately stimulating T-cell growth and then subsequently switching to the HTLV-I replication mode (Yoshida *et al*. [80]; Hinrichs *et al*. [81]). Of particular importance to the major thesis of this speculative paper is that the *rex *gene of HTLV-I interacts with the *rev *gene of HIV and this may suggest that the human virus may act to protect its host against some other pathogen.

## How HIV is 'Gentle' to its host

Some of the methods that HIV uses to be lysogenic and spread from host to host are obvious from its generally known biology (Daniel *et al*. [82]). Most obvious is that a retrovirus, in principle, would only be able to grow as the provirus in concert with a reproducing cell. HIV for example, can enter a quiescent cell and give rise to DNA products, but the DNA is only integrated after activation of the cell and replication of its chromosome (Zack *et al*., [83]; Stevenson *et al*., [84]; Haase *et al*., [85]). But the sole use of CD4 as a target receptor limits its opportunity to grow and reproduce. Maintenance of the latent state is another essential factor that keeps the virus from rapidly destroying the immune system. This is in addition to the host's immune system acting as an essential safeguard for viral long-term propagation within the host and transmission into other hosts.

## Conclusion

(a) The evolutionary mechanisms that maintain the non-virulent state of a pathogen are of high interest. Since there are a large number of mild pathogens in nature and in spite of the selective advantage that a virulent mutant would temporarily have, the 'gentle' pathogen state does arise and does persist. A proposed mechanism for this, not involving group or kin selection, has been made Koch [86]. This new model is that the pathogen protects itself (and incidentally its host) against other similar or identical pathogens arriving subsequently and that this generates and maintains the 'gentle' state.

(b) It is argued that pathogens that depend on humans as a resource faced a much different problem during the hunter/gatherer Stone Age than now, and at this early time the spectrum of viruses could be expected to have been largely STDs because of the sparse availability of hosts. Almost of necessity many pathogens must have been 'gentle' because the interactions between social groups were infrequent. Before moving to humans from the primates, HIV must have been gentle to its nonhuman primate host, and possibly it has not had time to readjust to become gentle its new host.

(c) Being a 'gentle' pathogen requires elaborate controls to self-limit growth of its host. Persistence over long times of a sexually transmitted disease depends, therefore, on growth inhibitory mechanisms in part coded by the virus, but frequently dependent on host function. The host immune system limits the viremia and viral encoded mechanisms and may act to modulate the immune response and act in other ways to control and limit viral growth. It can be assumed that the lentiviruses of nonhuman primates, such as SIV are adapted to a low rate of vertical transmission because of the devastating action of many viruses on neonates due to the latter's underdeveloped immune system. These may be avoided by slow growth. HIV is transmitted to offspring *in utero*, perinatally, or via breast-feeding, but the transmission is less efficient than for some other viral diseases. This suggests that host and viral mechanisms restrict vertical transmission or its effects for the case of HIV for the fetus and the neonate.

(d) The HIV retrovirus strategy depends on selectively infecting a restricted class of cells, mainly the CD4+ or CD8+ T helper cells. These and other T and B cells happen to be nearly the only suitable cells, *a priori*, in an mammal; these uniquely continue to grow, replicate, and divide to form progeny that remaining inside the host. Such a type of cell is the necessary condition for the strategy of retroviral growth. Thus these host cells are different than other cells of the body that only divide rarely or of cells that are shed from the animal such as skin and intestinal epithelium.

(e) It is proposed that the response of the mucosal part of the host immune system is not only the key factor to the prevention of many viral infections, but also it is to prevent or reduce the infection of the offspring of an infected female. This is because the selection for an ability to elicit an effective mucosal immune response by the products generated by the virus that would block secondary infection and is in the best interests of the virus, therefore. But it also makes the resident pathogen less destructive to its host and its host's progeny. I suggest that the role of mucosal immunological response and other defensive responses is critical for the biology of the STD retroviruses.

(f) The important link in the STD lifestyle as typified by the HIV/human interaction is that the virus must work against itself under conditions in which the hosts are at a premium and must somehow protect the fetus and neonate. It is the fact that such protection is manifest even with HIV infection of humans. About 85% of the offspring of HIV-infected mother are not HIV infected and in simian viruses in wild monkeys the number may be closer to 100%. Compared with some other viral diseases, this suggests that specific immune protection of the fetus perinatally does occur.

(g) The change from the forebears of HIV, presumed to be a 'gentle' virus of its non-human primates, to the devastating human virus of AIDS is mostly because the new host of the virus lives much longer than its previous host. Added to this are the factors due to the human host's social behavior, most notably the extensive movements of humans from place to place. Also HIV appears very ungentle now because of its spread to a greatly expanded habitat, and because of the sociology due to the homosexual involvement and intravenous drug use.
